# Baicalein protects rat insulinoma INS-1 cells from palmitate-induced lipotoxicity by inducing HO-1

**DOI:** 10.1371/journal.pone.0176432

**Published:** 2017-04-26

**Authors:** Hyun Jeong Kwak, Dongki Yang, Yongha Hwang, Hee-Sook Jun, Hyae Gyeong Cheon

**Affiliations:** 1 Department of Pharmacology, Gachon University College of Medicine, Incheon, Republic of Korea; 2 Department of Physiology, Gachon University College of Medicine, Incheon, Republic of Korea; 3 College of Pharmacy and Gachon Institute of Pharmaceutical Science, Gachon University, Incheon, Republic of Korea; 4 Gachon Medical Research Institute, Gil Medical Center, Incheon, Republic of Korea; Duke University School of Medicine, UNITED STATES

## Abstract

**Objective:**

β-Cell dysfunction plays a central role in the pathogenesis of type 2 diabetes (T2D), and the identification of novel approaches to improve β-cell function is essential to treat this disease. Baicalein, a flavonoid originally isolated from the root of *Scutellaria Baicalensis*, has been shown to have beneficial effects on β-cell function. Here, the authors investigated the molecular mechanism responsible for the protective effects of baicalein against palmitate (PA)-induced impaired β-cell function, and placed focus on the role of heme oxygenase (HO)-1.

**Methods:**

Rat pancreatic β-cell line INS-1 cells or mouse pancreatic islets were cultured with PA (500 μM) to induce lipotoxicity in the presence or absence of baicalein (50 μM), and the expressions of the ER stress markers, ATF-3, CHOP and GRP78 were detected by Western blotting and/or qPCR. The involvement of HO-1 was evaluated by HO-1 siRNA transfection and using the HO-1 inhibitor ZnPP.

**Results:**

Baicalein reduced PA-induced ER stress and inflammation and enhanced insulin secretion, and these effects were associated with the induction of HO-1. Furthermore, these protective effects were attenuated by ZnPP and by HO-1 siRNA. Pretreatment of PD98059 (an ERK inhibitor) significantly inhibited the protective effects of baicalein and blocked HO-1 induction. On the other hand, CO production by RuCO (a CO donor) ameliorated PA-induced ER stress, suggesting that CO production followed by HO-1 induction may contribute to the protective effects of baicalein against PA-induced β-cell dysfunction.

**Conclusion:**

Baicalein protects pancreatic β-cells from PA-induced ER stress and inflammation via an ERK-HO-1 dependent pathway. The authors suggest HO-1 induction in pancreatic β-cells appears to be a promising therapeutic strategy for T2D.

## Introduction

Pancreatic β-cell dysfunction plays a central role in the development of diabetes mellitus, and it has been documented that chronic exposure to elevated free fatty acid (FFA) levels causes β-cell dysfunction and apoptosis [1]. Although the mechanism of lipotoxicity has not been fully clarified, accumulating evidence suggests the induction of endoplasmic reticulum (ER) stress might contribute to the detrimental effects of FFA on β-cells [2]. ER is a specialized organelle responsible for the synthesis, initial posttranslational modification, folding, and export of secretion of membrane proteins. When ER homeostasis is disrupted by biochemical, physiological, or pathological stimuli, unfolded or misfolded proteins accumulate in ER lumen, overwhelm ER chaperons, such as, glucose-regulated protein 78 (GRP78), and cause ER stress. Moderate ER stress may represent a defense mechanism against external stimuli, but excessive or persistent ER stress eventually triggers programmed cell death or apoptosis by activating the caspase-12, C/EBP homology protein (CHOP), or the c-Jun-N-terminal kinase (JNK)-dependent pathway [3]. Recently, considerable evidence has been presented that suggests ER stress contributes to FFA-induced β-cell dysfunction by upregulating pro-apoptotic effectors, such as, CHOP [4]. Consequently, pharmacological agents that alleviate ER stress may provide novel means of protecting β-cells from lipotoxicity.

Baicalein is a flavonoid that exerts broad pharmacological effects, including anti-inflammatory and antioxidant effects, and was originally isolated from the roots of *Scutellaria Baicalensis* [5]. Recently, much interest has been focused on the antidiabetic effects of baicalein. In particular, it has been demonstrated that baicalein improves hyperglycemia and glucose intolerance, and promotes insulin secretion by inhibiting islet cell apoptosis in streptozotocin-induced diabetic mice [6].

Heme oxygenase-1 (HO-1) is an anti-inflammatory molecule that catalyzes heme breakdown to carbon monoxide, biliverdin, and iron, and is induced by numerous stressors including endotoxin, cytokines, and oxidants [7]. In humans and mice, loss of HO-1 function in Kupffer cells and PBMCs leads to early death due to increased susceptibility to inflammation [8–10]. Interestingly, several studies have shown that chemical inducers of HO-1 ameliorate obesity and diabetes in different models [11]. Furthermore, efforts to identify pharmacological or natural agents with HO-1-inducing ability were initiated after it was reported HO-1 induction in islets afforded protection from the detrimental effects of pro-inflammatory cytokines [12]. The iron produced by the catalytic breakdown of HO-1 produces ROS, and bilirubin has been reported to have both cytotoxic and cytoprotective effects [13, 14]. However, CO has vasodilatory effects, and inhalation of CO has been reported to protect tissues against hyperoxia and to reduce obesity, hyperglycemia, and insulin resistance [15–17]. Based on previous findings regarding the role of HO-1, we investigated whether baicalein protects β-cells from PA-induced ER stress and inflammation using INS-1 cells, and subsequently sought to determine whether its protective effects are associated with HO-1 induction.

## Materials and methods

### Chemicals

RPMI 1640 medium, fetal bovine serum (FBS), fetal calf serum (FCS), penicillin, and streptomycin were obtained from GIBCO (Grand Island, NY). Antibodies against ATF-3, CHOP, GRP78, HO-1, ERK, pERK, and β-actin were from Santa Cruz Biotechnology (Santa Cruz, CA). The RNA extraction kit was from Intron Biotechnology (Seoul, Korea). Rat oligonucleotide primers for ATF-3, GRP78, CHOP, HO-1, GAPDH, and rat HO-1 siRNA were from Bioneer Co. Ltd (Daejeon, Korea). PD98059 was from Calbiochem-Novabiochem Co. (San Diego, CA). Baicalein, tricarbonyldichloro-ruthenium(II) dimer (RuCO), cobalt protoporphyrin IX (CoPP), zinc protoporphyrin IX (ZnPP) and all other chemicals were from Sigma-Aldrich (St Louis, MO).

### Cell culture and treatment

INS-1 cells (a rat pancreatic β-cell line) were obtained from the Korean cell line bank (Seoul, Korea) and cultured in RPMI 1640 medium containing 25 mM HEPES, 2 mM glutamine, 1 mM pyruvate (Gibco, Carlsbad, CA) and 10% FBS at 37°C in a 5% CO_2_ atmosphere_._ For experiments, INS-1 cells (5x10^4^ cells/ per well) were treated with baicalein (50 μM) for 3 h in the absence or presence of ZnPP (10 μM) or PD98059 (10 μM). PA (500 μM) was then added and culture continued for 8 h (for qPCR) or 24 h (for western blot). Separately, INS-1 cells were pretreated with CoPP (10 μM) for 1 h and then with PA for either 8 h or 24 h.

### Preparation of pancreatic islets

Islets were isolated from 9-week-old male C57BL/6 mice (Orient bio, Kyounggi-do, Korea) using a collagenase digestion method. Briefly, after injecting 3 ml of liberase (0.17 mg/ml, Roche, Basel, Switzerland) into bile ducts, each swollen pancreas was excised and incubated in a water bath at 37°C for 20 min. After stopping the liberase digestion with cold Hanks' balanced salt solution (HBSS, Sigma, St. Louis, MO), pancreatic tissues were disrupted by bubble pipetting. Islets were separated by centrifugation on 25%, 23%, 21.5%, 20.5% and 11.0% Ficoll (Sigma, St. Louis, MO) gradients. Islets at the interface between the 21.5% and 11.0% fractions were collected and washed with HBSS. Healthy islets were hand-picked under a stereomicroscope (Leica, Germany). Isolated islets were maintained in RPMI 1640 medium containing 5.5 mM glucose for 24 h, and then, the cells were treated with baicalein (50 μM) for 3 h in the absence or presence of ZnPP (10 μM) followed by the addition of PA (500 μM) for further 8 h for qPCR.

### FFA preparation

Palmitic acid (Sigma-Aldrich) was dissolved in 100% EtOH to a concentration of 100 mM, heated to 70°C, and diluted in RPMI medium containing freshly prepared 2% fatty acid free BSA.

### Cell viability assay

Cell viabilities were determined using a 3-[4,5-2-yl]-2,5-diphenyltetrazolium bromide (MTT) assay. Subconfluent INS-1 cells in 24-well plates were incubated with various concentrations of baicalein for 3 h and then exposed to PA (500 μM) for 8, 24, or 48 h. MTT solution (100 μl/well; 5 mg/ml in PBS) was then added and incubation continued for 3 h. The medium was then replaced with dimethylsulfoxide (DMSO). Plates were shaken for 20 min and optical densities (OD) were measured at 570 nm using a microplate reader (Victor 4; Perkin Elmer, Waltham, MA).

### Apoptosis assay

DNA fragmentation activity with INS-1 cells was quantified using a Cell Death Detection ELISA Plus kit (Roche Diagnostics GmbH, Mannheim, Germany) according to the manufacturer’s instructions. For this assay, cells were treated with various concentrations of baicalein (10, 50, and 100 μM) for 3 h before exposure to PA (500 μM) for 24 h in the presence or absence of ZnPP. Following treatment, the cells were washed twice with PBS and incubated with lysis buffer for the extraction of histone fragment at room temperature. Following centrifugation to remove the nuclei and cellular debris, the supernatants (containing histone fragment) were incubated in microtiter plates that were coated with anti-histone antibody and detected by peroxidase-conjugated antibody. Color was developed with an ABTS (2,21-azino-bis(3-ethylbenzothiazoline-6-sulphonic acid) substrate, which was read at 405 nm using Victor 4 Elisa reader (Perkin Elmer, Waltham, MA).

### Transfection of small interfering RNAs (siRNAs)

Rat HO-1 siRNA duplexes were designed and synthesized by Bioneer Co. Ltd. (Daejeon, Korea). The sequences used were as follows: rat HO-1 (GenBank: NM_012580.2), 5′-ACAAGCAGAACCCAGUCUA-3 (F), 5-UAGACUGGGUUCUGCUUGU-3’ (R). For HO-1 knockdown, INS-1 cells (5×10^5^ cells/well) were transfected with either HO-1 siRNA (20 nM) or control siRNA using the RNAimax-transfection system (Invitrogen, Carlsbad, CA). After transfection for 36 h, cells were treated with PA in the presence of baicalein (50 μM) for 12 h (for qPCR) or 24 h (for Western blot).

### Protein extraction and western blotting

Cells were incubated as described in cell culture and treatment section, lysed in PRO-PREP™ protein extraction solution (Intron Biotechnology), and then incubated for 30 min at 4°C. Protein contents were determined using Bio-Rad protein assay reagent. Equal amounts of protein were loaded on 10% SDS gels and semidry transferred to 0.45 μm nitrocellulose membranes (Biostep, Jahnsdorf, Germany), which were treated with primary antibodies against GRP78, CHOP, ATF-3, and HO-1, incubated overnight at 4°C, washed four times with Tween 20/Tris-buffered saline (T-TBS), and then incubated with horseradish peroxidase-conjugated secondary antibody (1:1000) for 1 h at room temperature. Specific bands were detected using a chemiluminescence kit (Amersham Life Science, Buckinghamshire, UK). Band densities were quantified using Image J software.

### RNA extraction and quantitative real time PCR (qRT-PCR)

RNA was extracted using Easy Blue® kits (Intron Biotechnology). RNA (1 μg) was reverse transcribed using TOPscript RT DryMix (Enzynomics, Daejeon, Korea). SyberGreen analyses were performed using qPCR mastermix (Toyobo, Japan). The rat primers used were as follows; GRP78: 5′-CCTATTCCTGCGTCGGTGTATT-3′ (F) and 5′-GGTTGGACGTGAGTTGGTTCT-3′ (R), CHOP: 5′-GAAATCGAGCGCCTGACCAG-3′ (F) and 5′-GGAGGTGATGCCAACAGTTCA-3′ (R), ATF-3: 5′-GCTGCCAAGTGTCGAAACAAGA-3′ (F) and 5′-GTGCAGGTTGAGCATGTAAATCAGA-3′ (R), HO-1: 5′-TTGTCTCTCTGGAATGGAAGG -3′ (F) and 5′-CTCTACCGACCATTCTG -3′ (R), GAPDH: 5′-CCCCAATGTATCCGTTGTGGA-3′ (F) and 5′- GCCTGCTTCACCACCTTCTT-3′ (R). Mouse-GRP78: 5′-CTGGACTGAATGTCATGAGGATCA-3′ (F) and 5′-CTCTTA TCC AGG CCATATGCAATAG-3′ (R), ATF3: 5′-AACTGGCTTCCTGTGCACTT-3′ (F) and 5′-TGAGGCCAGCTAGGTCATCT-3′ (R), CHOP: 5′- CCACCACACCTGAAAGCAGAA-3′ (F) and 5′-GGTGCCCCCAATTTCATCT-3′ (R), HO-1: 5′-GATAGAGCGCAACAAGCA GAA-3′ (F) and 5′- CAGTGAGGCCCATACCAGAAG-3′ (R), GAPDH: 5′-CTCAACTACA TGGTCTACATG TTCCA-3′ (F) and 5′-CCATTCTCGGCCTTGACTGT-3′ (R). The mRNA levels were determined using a Roche Light cycler 2.0 (Roche Bio Inc., Switzerland), and results were normalized versus GAPDH.

### Measurement of insulin secretion

INS-1 cells were plated in 24-well plates (5x10^4^ cells/ per well) and incubated with baicalein (50 μM) in the presence or absence of 500 μM PA for 24 h. The culture medium was then removed, and the cells were washed and preincubated in Krebs−Ringer buffer (KRB; 135 mM NaCl, 3.6 mM KCl, 0.5 mM NaH_2_PO_4_, 0.5 mM MgSO_4_, 1.5 mM CaCl_2_, 2 mM NaHCO_3_, 10 mM HEPES, and 0.1% w/v BSA, pH 7.4) without glucose for 1 h at 37°C. Thereafter, the cells were incubated in KRB containing 25 mM glucose for 1 h at 37°C. Supernatants were collected and insulin levels were measured using an insulin ELISA kit (Millipore, Bedford, MA) according to the manufacturer’s protocol.

### Measurement of cytokine levels

Cytokine levels in culture medium were measured by enzyme-linked immunosorbent assay (ELISA) using an OptEIA mouse TNF-α and IL-6 kits (BD Pharmingen, Seoul, Korea), according to the manufacturer’s instructions. Absorbance was measured at 405 nm with spectrophotometer (Victor 4).

### Statistical analyses

The experimental results are presented as means ± SDs of three separate experiments. One-way ANOVA with a Tukey’s *post hoc* analysis was used for the comparison of data among the experimental groups, and *p* values of < 0.05 were considered statistically significant. Statistical analysis was performed using the SPSS18.0 software (SPSS Inc., Chicago. IL).

## Results

### Effects of baicalein on PA-induced cytotoxicity and ER stress in INS-1 cells

**Fig 1:**
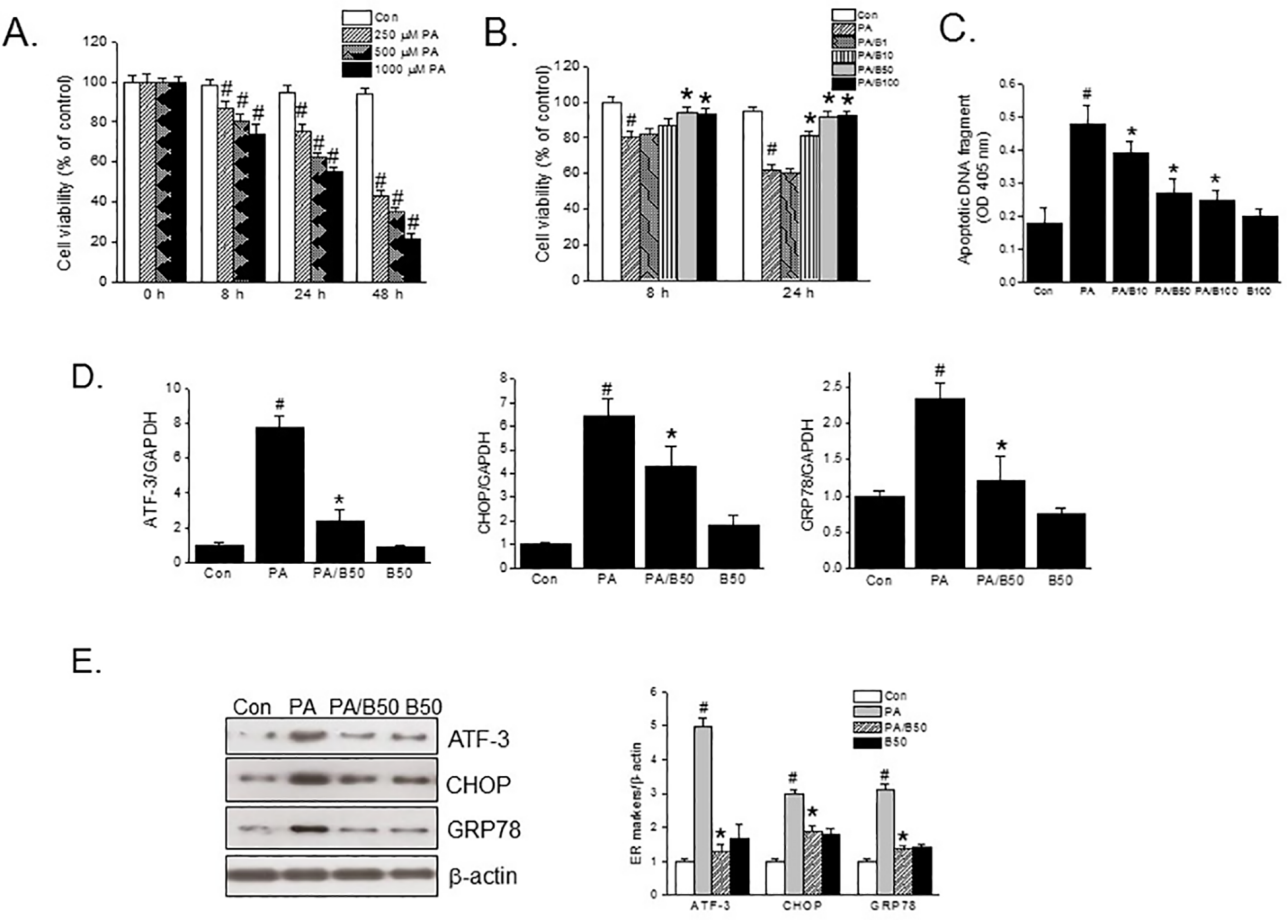
Effects of baicalein on PA-induced cytotoxicity and ER stress. INS-1 cells were treated with various concentrations of PA (0–1000 μM) for different times (0–48 h) (A). INS-1 cells were treated with different concentrations of baicalein (0–50 μM) for 3 h and then PA (500 μM) was added and incubation continued for a further 8 h or 24 h (B). Cell viabilities were assessed using a MTT assay, and the results are shown as % of control (2% BSA treatment at 0 h). Apoptotic DNA fragmentation was assessed using a Cell Death Detection ELISA Plus kit, and the results are shown as OD at 405 nm (C). INS-1 cells were pretreated with baicalein (50 μM) for 3 h and then PA (500 μM) was added and incubation continued for a further 8 h (for qPCR) or 24 h (for western blot). The mRNA expressions (D) and protein (E) levels of ER stress markers were assessed by qPCR or western blotting. The results shown are representative of three independent experiments and are expressed as means ± SDs. ^#^P<0.05 vs. controls; *P<0.05 vs. PA alone.

To check the effect of PA on INS-1 cell viability, various concentrations of PA were added to INS-1 cells for different incubation times. As shown in Fig 1A, PA reduced cell viability in a concentration and time dependent manner. Strikingly, PA induced cell death by more than 50% after incubation for 48 h regardless of its concentration. On the other hand, pretreatment with baicalein for 3 h protected cells from PA (500 μM)-induced cell death to almost the control level at concentrations of 50 μM or higher (Fig 1B). In addition, the reversal of PA-induced cell death by baicalein was associated with anti-apoptotic effects since baicalein reduced PA-induced apoptosis (Fig 1C).

It has been shown ER stress contributes to FFA-induced β-cell dysfunction [2]. Thus, we examined the effects of baicalein on PA-induced ER stress. Fig 1D and 1E showed that baicalein pretreatment (50 μM) significantly reduced the levels of ATF-3, CHOP, and GRP78 mRNAs and proteins (all markers of ER stress) induced by PA. MTT assays showed baicalein itself had no obvious cytotoxic effect on INS-1 cells at concentrations up to 100 μM (data not shown).

### Baicalein protected cells from PA-induced ER stress via a HO-1 dependent mechanism

**Fig 2:**
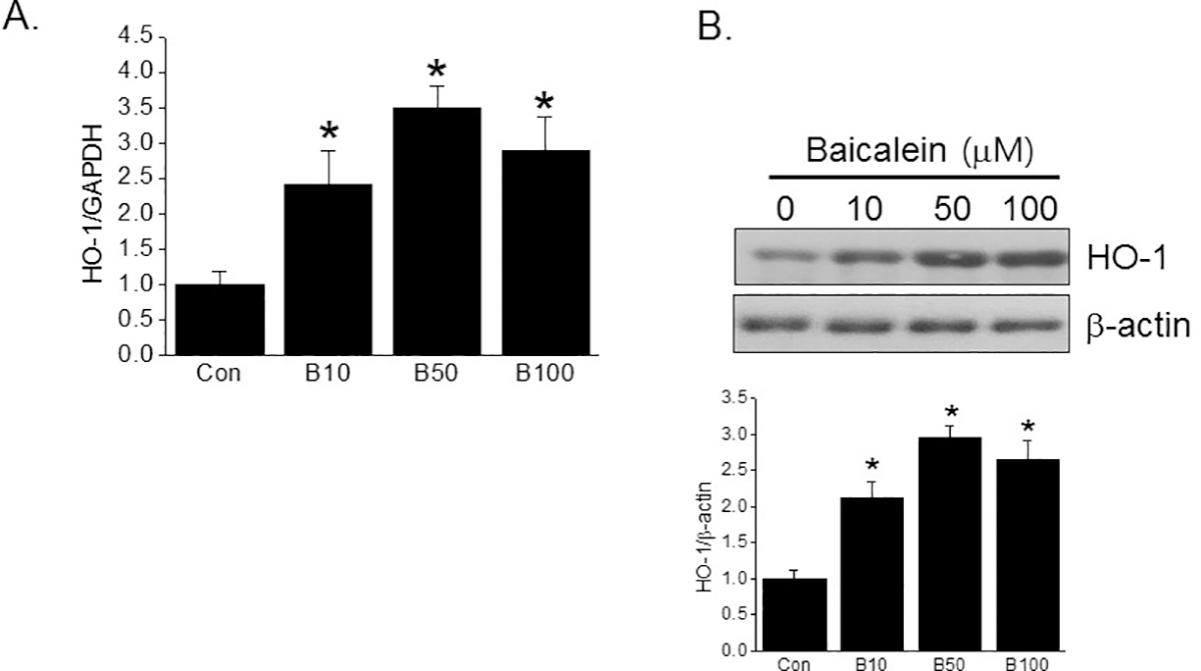
Effects of baicalein on HO-1 expression. INS-1 cells were treated with different concentrations of baicalein (0–100 μM) for 11 h (A, for qPCR) or 27 h (B, for western blot). HO-1 mRNA expression and protein levels were assessed by qPCR or western blotting, respectively. The results shown are representative of three independent experiments and are expressed as means ± SDs. *P<0.05 vs. controls.

**Fig 3:**
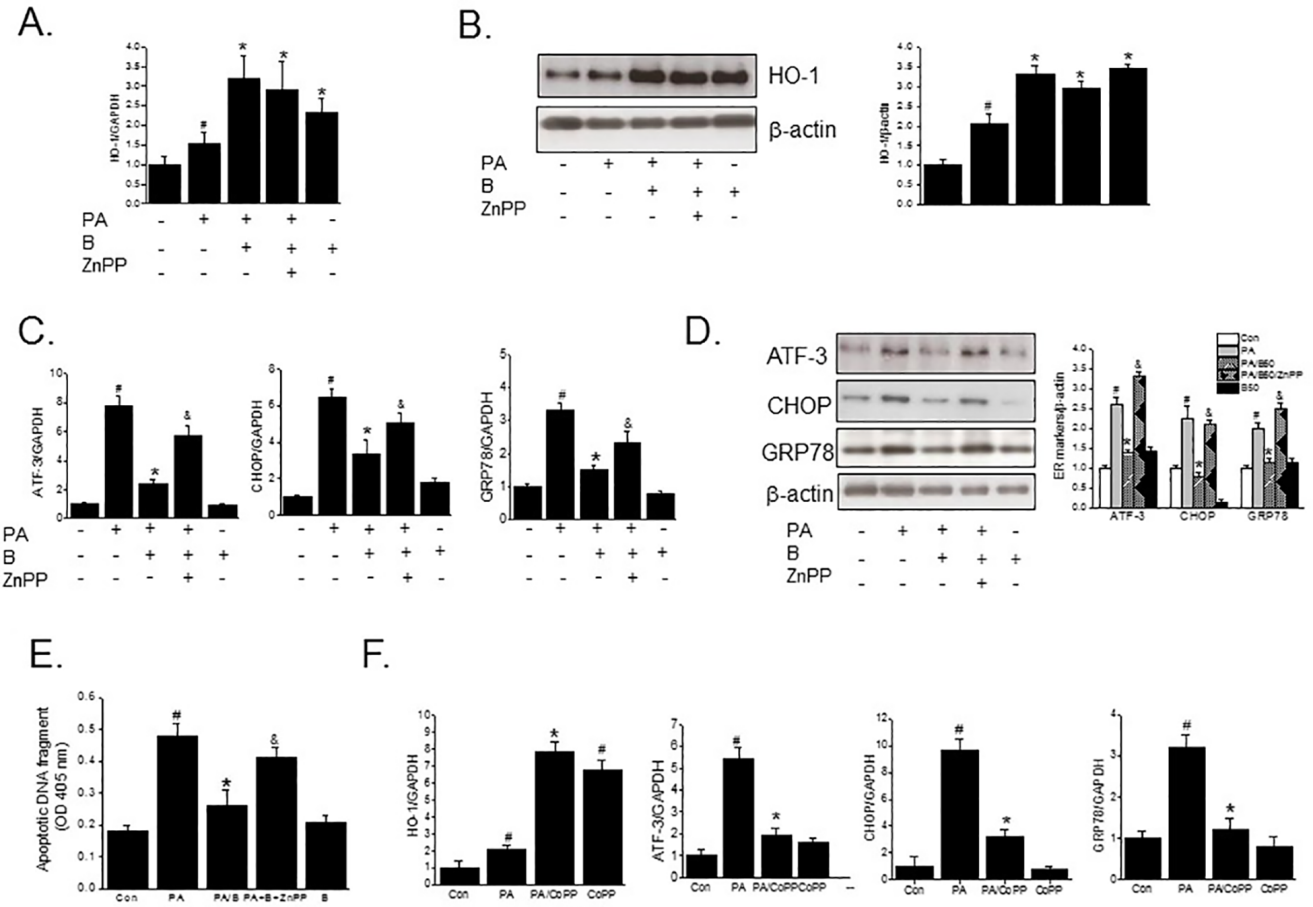
Involvement of HO-1 upregulation in the protective effect of baicalein on PA-mediated lipotoxicity. INS-1 cells were pretreated with or without ZnPP (10 μM) for 30 min, followed by baicalein (50 μM) for 3 h and then PA (500 μM) for a further 8 h (for qPCR) or 24 h (for western blot). The mRNA expressions (A and C) and protein (B and D) levels of HO-1 or ER stress markers were assessed by qPCR or western blotting, respectively. Apoptotic DNA fragmentation was assessed using a Cell Death Detection ELISA Plus kit (E). INS-1 cells were pretreated with CoPP (10 μM) for 1 h, followed by PA (500 μM) for a further 8 h, and mRNA expressions were determined by qPCR (F). The results shown are representative of three independent experiments and are expressed as means ± SDs. ^#^P<0.05 vs. controls; *P<0.05 vs. PA alone; ^&^P<0.05 vs. PA plus baicalein treatment.

To explore the mechanism underlying the protective effect of baicalein, we focused on the effect of baicalein on HO-1 induction in INS-1 cells, as previous reports have shown that baicalein induces HO-1 expression in macrophages and cardiomyocytes [18, 19]. As shown in Fig 2A and 2B, baicalein enhanced HO-1 expression maximally at 50 μM. To confirm HO-1 plays an important role in the protective effect of baicalein against PA-induced lipotoxicity, cells were pretreated with ZnPP (a widely used HO-1 inhibitor). Whereas ZnPP had little effect on baicalein-induced HO-1 expression (Fig 3A and 3B), the protective effects of baicalein against PA-induced ER stress and apoptosis were abrogated by the addition of ZnPP (10 μM) (Fig 3C–3E). To evaluate the involvement of HO-1 in the protective effects of baicalein, cells were pretreated with CoPP (a HO-1 inducer) to examine its effect on PA-induced lipotoxicity. As shown in Fig 3F, CoPP (10 μM) pretreatment completely suppressed PA-induced ER stress marker expression. These results imply that the protective effects of baicalein indeed appear to be mediated through HO-1 induction.

**Fig 4:**
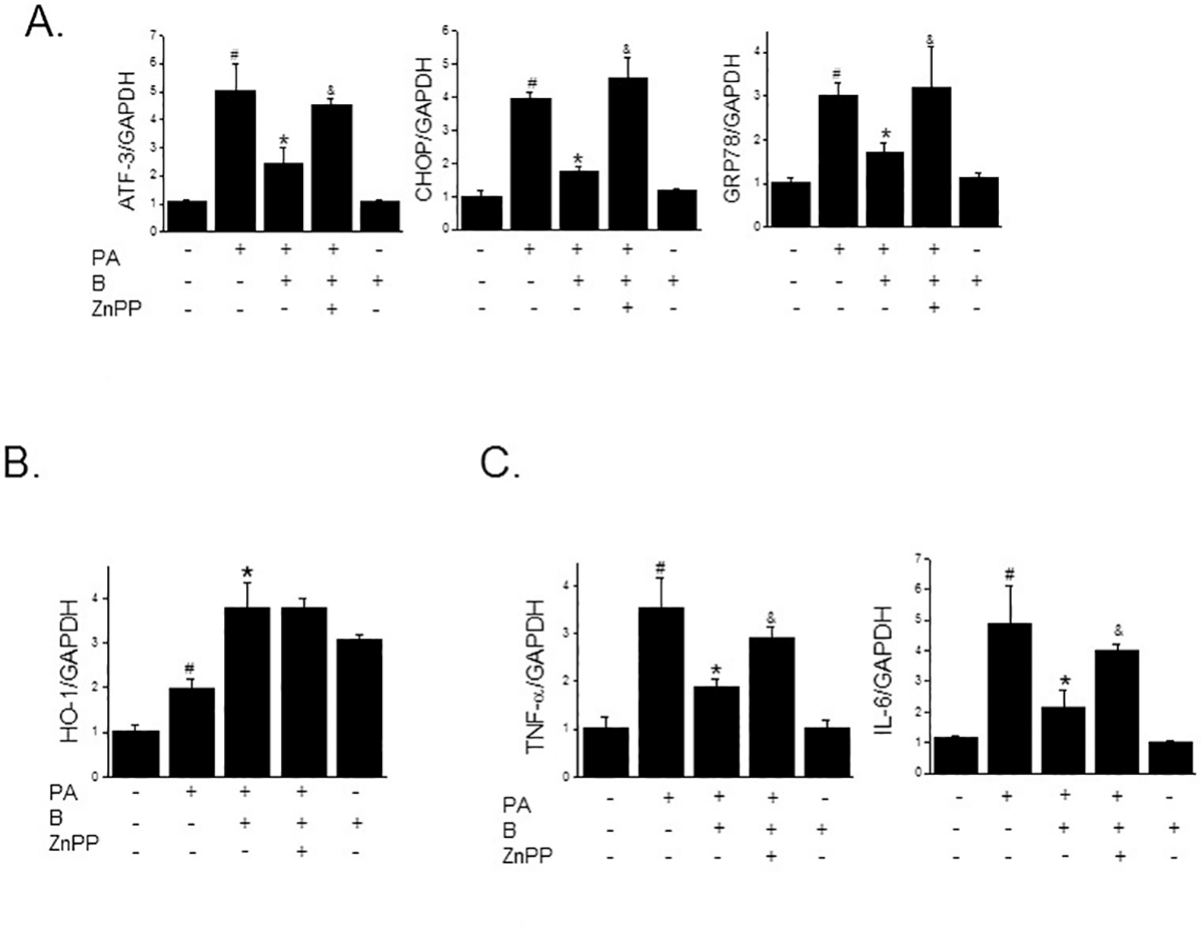
Effects of baicalein on PA-induced lipotoxicity in isolated pancreatic islets. Pancreatic islets isolated from C57BL/6J mouse (9 weeks old, male) were pretreated with or without ZnPP (10 μM) for 30 min, followed by baicalein (50 μM) for 3 h and then PA (500 μM) for a further 8 h. The mRNA expressions of HO-1 (B) or ER stress (A) and proinflammatory markers (C) were assessed by qPCR. The results shown are representative of three independent experiments and are expressed as means ± SDs. ^#^P<0.05 vs. controls; *P<0.05 vs. PA alone; ^&^P<0.05 vs. PA plus baicalein treatment.

To further confirm the protective effects of baicalein against PA-induced ER stress, pancreatic islets were isolated from C57BL/6 mice, and treated with PA in the presence or absence of baicalein. Similar to the effects on INS-1 cells, baicalein reduced PA-induced ER stress and inflammation, and its effects were reversed by ZnPP, confirming that the protective effect of baicalein against PA-induced ER stress is mediated by HO-1 induction (Fig 4).

### ERKs pathway participated in the protective action of baicalein against PA-induced ER stress

**Fig 5:**
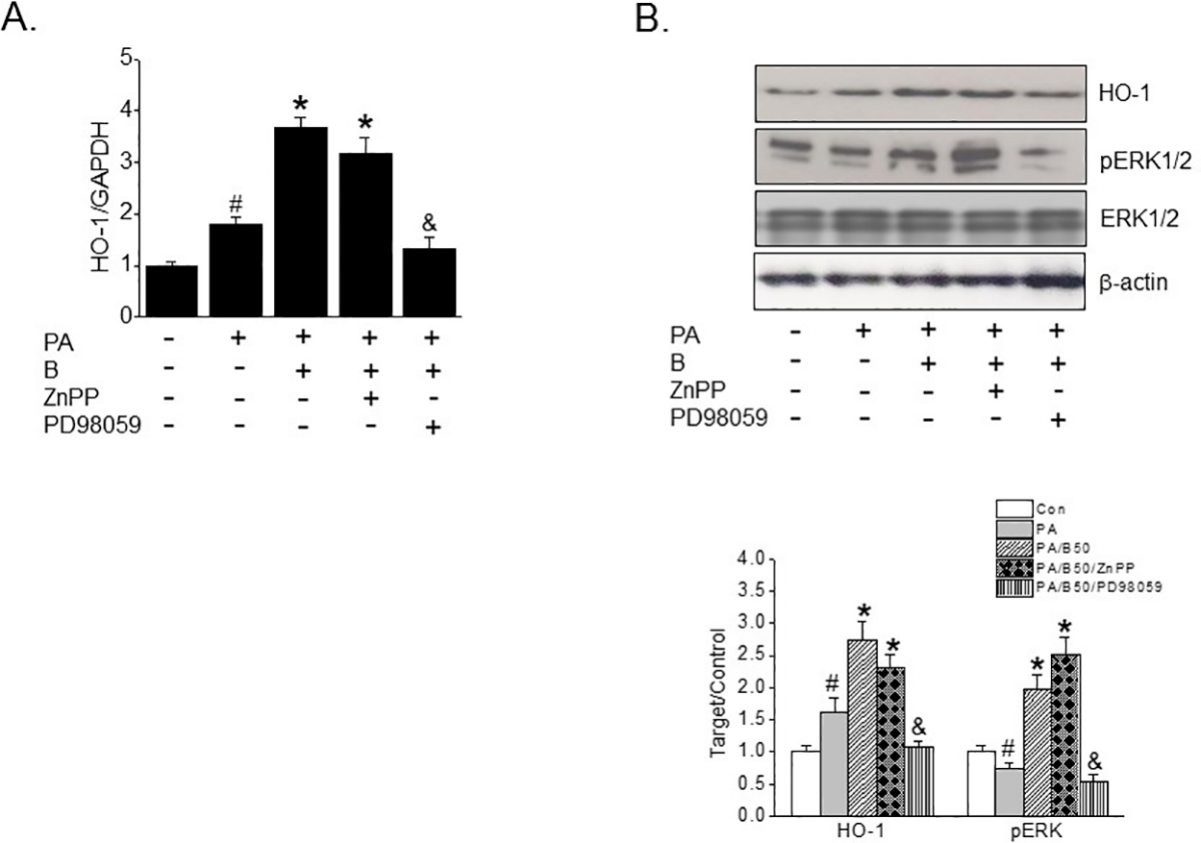
Effects of ERK activation on baicalein-induced HO-1 expression. INS-1 cells were pretreated with baicalein (50 μM) for 3 h in the absence or presence of ZnPP (10 μM), or PD98059 (10 μM) and then PA (500 μM) was added and incubation continued for a further 8 h (for qPCR) or 24 h (for western blot). The mRNA expression of HO-1 (A) and protein levels of HO-1, pERK1/2 and ERK1/2 (B) were assessed by qPCR or western blotting, respectively. Results are representative of three independent experiments and are presented as means ± SDs. ^#^P<0.05 vs. controls; *P<0.05 vs. PA alone; ^&^P<0.05 vs. PA plus baicalein treatment.

It has been well established that the activations of intracellular kinases, such as, MAPKs, are associated with the expressional regulation of several genes [20]. Thus, we examined whether MAPK activation is required for the baicalein-mediated inductions of HO-1 mRNA and protein. As shown in Fig 5, when INS-1 cells were co-treated with PD98059 (10 μM, an ERK inhibitor) and baicalein, baicalein-induced HO-1 mRNA and protein inductions were markedly inhibited with reduced ERK phosphorylation. Neither SB203580 (10 μM; a p38 MAPK inhibitor) nor SP600125 (10 μM; a JNK inhibitor) elicited an inhibitory effect on baicalein-mediated HO-1 induction (results not shown). In addition, ERK phosphorylation was unaffected by ZnPP (10 μM; a HO-1 inhibitor) co-treatment. These findings confirmed ERK phosphorylation is an upstream event of baicalein-induced HO-1 expression in INS-1 cells.

### Baicalein ameliorated insulin secretion and reduced inflammatory cytokine levels through an ERK-HO-1 dependent pathway

**Fig 6:**
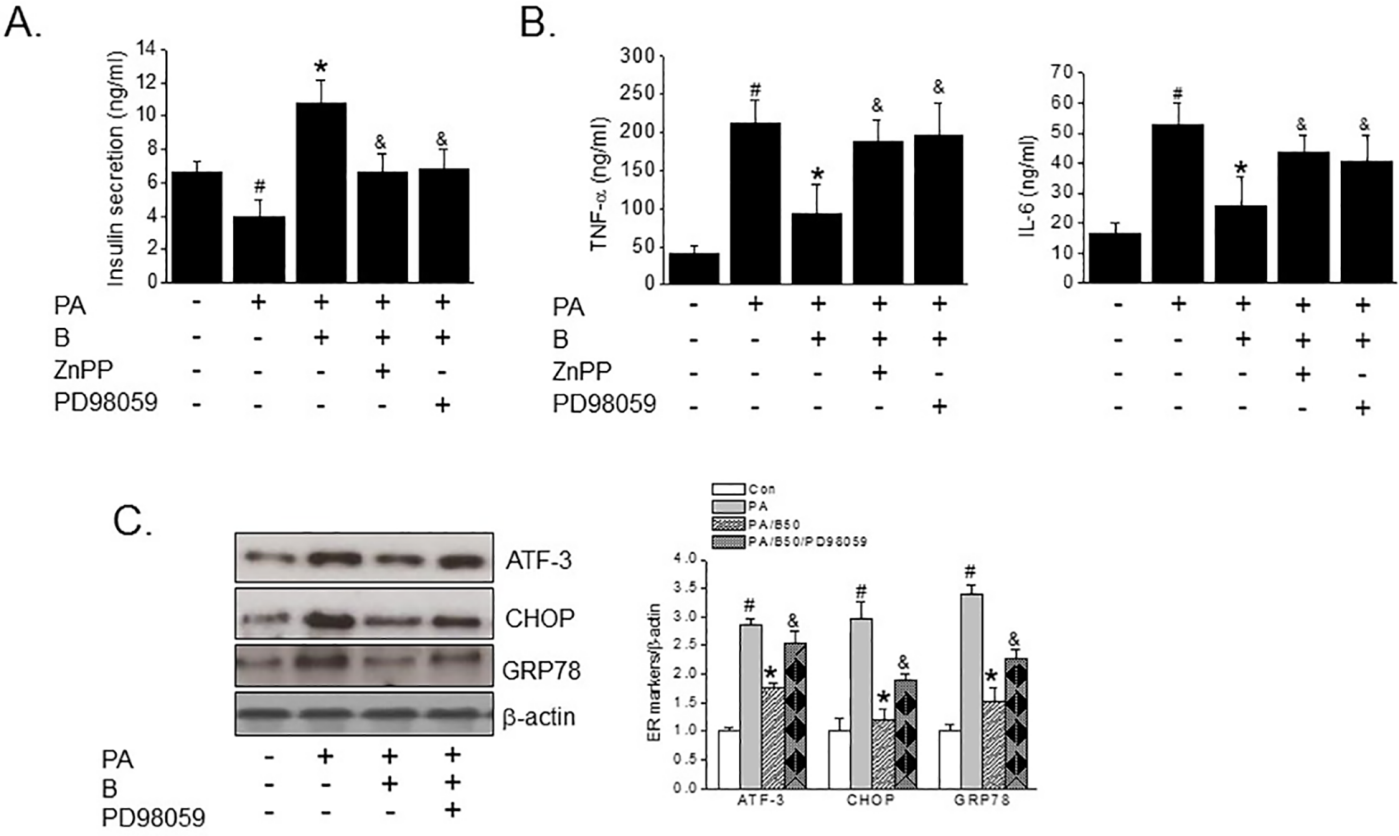
Effects of baicalein on PA-induced inflammation and reduced insulin secretion. INS-1 cells were pretreated with either ZnPP (10 μM) or PD98059 (10 μM) for 30 min, followed by baicalein (50 μM) for 3 hr. And then PA (500 μM) was added and further incubated for 24 h. To examine glucose-stimulated insulin secretion, culture medium was replaced with medium containing 25 mM glucose for 1 h, and then insulin secretion to medium was measured by ELISA (A). INS-1 cells were pretreated with baicalein (50 μM) for 3 h in the absence or presence of ZnPP (10 μM), or PD98059 (10 μM) and then PA (500 μM) was added and incubation continued for a further 8 h (for qPCR) or 24 h (for western blot). Levels of TNF-α and IL-6 were assessed by ELISA (B). The protein levels of ER stress markers were determined by Western blotting (C). The results shown are representative of three independent experiments and are expressed as means ± SDs. ^#^P<0.05 vs. controls; *P<0.05 vs. PA alone; ^&^P<0.05 vs. PA plus baicalein treatment.

In order to confirm further that HO-1 induction and ERK activation are involved in the protective effect of baicalein against PA-induced dysfunction in INS-1 cells, we examined the effects of baicalein on the PA-induced impairment of insulin secretion. Baicalein prevented the reduction in glucose-stimulated insulin secretion induced by PA, and reduced PA-induced increases in pro-inflammatory cytokine levels, including TNF-α and IL-6 levels. Furthermore, these beneficial effects of baicalein were substantially blocked when ZnPP or PD98059 were co-treated with baicalein (Fig 6A and 6B). In addition, the protective effects of baicalein against PA-induced ER stress were diminished when PD98059 was co-treated with baicalein (Fig 6C). These observations imply that the ERK-HO-1 pathway is required for the protective effect of baicalein against PA exposure in INS-1 cells.

### Loss of HO-1 expression abolished the protective effects of baicalein

**Fig 7:**
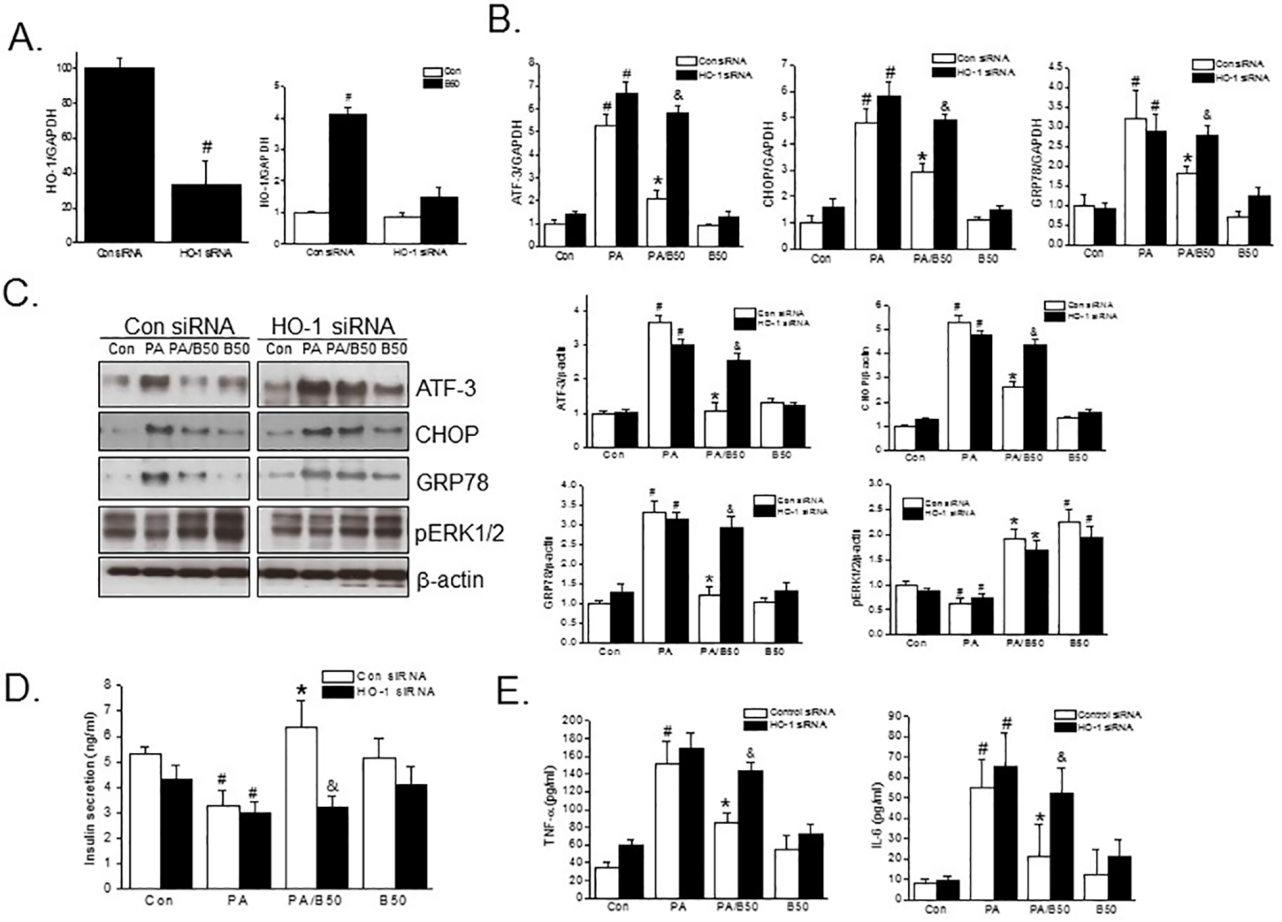
Attenuation of the protective effects of baicalein by HO-1 knockdown. INS-1 cells were transfected with 20 nM HO-1 siRNA or control siRNA for 36 h, and the reduction of HO-1 mRNA expression was determined (A left). HO-1 siRNA or control siRNA transfected cells were treated with baicalein (50 μM) for 3 h, and then HO-1 mRNA expression was determined by qPCR (A right). After HO-1siRNA transfection, cells were treated with baicalein (50 μM) for 3 h and PA was then added and incubation continued for 8 h (for qPCR) or 24 h (for western blot or ELISA). The expression levels of ER stress makers (B) were assessed by qPCR. Protein levels of ER stress markers and phosphorylated ERKs were determined by Western blotting (C). Insulin secretion (D) and secreted TNF-α and IL-6 (E) levels were assessed by ELISA. The results shown are representative of three independent experiments and are expressed as means ± SDs. ^#^P<0.05 vs. controls; *P<0.05 vs PA alone; ^&^P<0.05 vs. PA plus baicalein with control siRNA transfection.

The effect of HO-1 knockdown was examined to confirm further the involvement of HO-1 in the protective effect of baicalein against PA-induced lipotoxicity in INS-1 cells. As shown in Fig 7A, HO-1 siRNA transfection markedly reduced HO-1 expression by 66.8 ± 5.3% vs. control siRNA, and the induction of HO-1 by baicalein was prevented by HO-1 knockdown. Concomitantly, the suppressive effect of baicalein on the mRNA and protein expressions of ER stress markers disappeared after HO-1 knockdown (Fig 7B and 7C). However, the phosphorylation of ERK by baicalein was not diminished by HO-1 knockdown, which again suggested that ERK phosphorylation is an upstream signal for HO-1 induction (Fig 7C). Consistent with the involvement of HO-1 in baicalein action, knockdown of HO-1 in INS-1 cells blocked increased insulin secretion and decreased TNF-α and IL-6 production by baicalein (Fig 7D and 7E). These findings confirm HO-1 induction is critically involved in the protective effect of baicalein against PA-induced lipotoxicity in INS-1 cells.

### Carbon monoxide was involved in the protective effect of baicalein against PA-induced lipotoxicity

**Fig 8:**
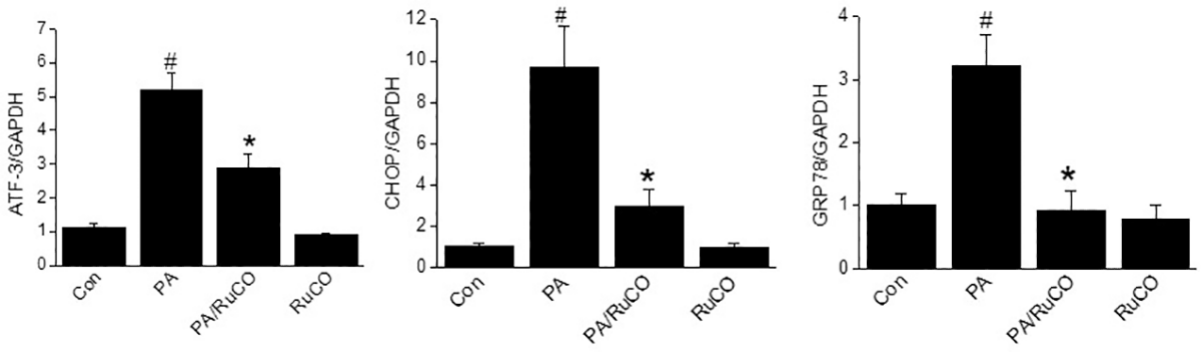
Effects of RuCO (a CO donor) on PA-mediated lipotoxicity. INS-1 cells were treated with RuCO (150 μM) for 1 h and then incubated with PA (500 μM) for 8 h. The mRNA expression levels of ER stress markers were detected by qPCR. The results shown are representative of three independent experiments and are expressed as means ± SDs. ^#^P<0.05 vs. controls; *P<0.05 vs PA alone.

HO-1 catalyzes the cleavage of heme, to produce CO, biliverdin, and iron. We examined whether CO participated in the amelioration of PA-induced lipotoxicity by pretreating INS-1 cell with the CO donor RuCO before PA treatment. As shown in Fig 8, RuCO (150 μM) pretreatment significantly inhibited the PA-induced expressions of ATF-3, CHOP and GRP78 in INS-1 cells. Together, these results suggest CO may play an important role in the protective effect of baicalein against PA-induced dysfunction in INS-1 cells.

## Discussion

The findings reported herein suggest baicalein protects rat pancreatic INS-1 cells from PA-induced ER stress and inflammation and improves insulin secretion via HO-1 induction. In addition, it was shown ERK activation appears to be an upstream signal of baicalein-induced HO-1 expression and that HO-1 catalyzed CO production plays a key role in the protective effect of baicalein.

It is well known β-cell dysfunction is involved in the development of diabetes mellitus, and that lipotoxicity importantly contributes to β-cell dysfunction, and thus to the pathogenesis of T2D [21]. Prolonged exposure of pancreatic β-cells to the elevated levels of FFA causes β-cell apoptosis, possibly via the induction of ER stress [2]. Indeed, ER plays an important role in β-cell homeostasis because insulin synthesis begins in the ER, where pre-proinsulin is folded and then cleaved to form proinsulin, which subsequently matures to insulin in Golgi. Furthermore, perturbed insulin biosynthesis in the ER leads to β-cell dysfunction and impairs glucose homeostasis. Accordingly, protection from PA-induced β-cell dysfunction using small molecules or natural products offers an attractive strategy for T2D. Our results show baicalein can ameliorate PA-induced lipotoxicity by alleviating cell death and ER stress and protect β-cell functions as indicated by its protection of INS-1 cells from lipotoxicity-induced reduction of insulin secretion.

Baicalein has been previously shown to have beneficial effects on diabetes related-complications [6, 22, 23]. In particular, its efficacy against diabetic retinopathy was attributed to its inhibition of p38 MAPKs expression and oxidative stress [24]. Notably, baicalein was found to activate AMPK, which may be implicated in the attenuation of insulin resistance in diabetic mice [25]. Furthermore, AMPK activation in liver repressed fatty acid and cholesterol synthesis by decreasing the activations of SREBP-1c and FAS and increasing ACC phosphorylation [25]. In accordance with *in vivo* data, baicalein has also been shown to promote the survival of insulin secreting cells [6]. However, the mechanisms involved in the protective effects of baicalein action have not been clearly defined.

HO-1 has been shown to play an important role in redox homeostasis and in cellular defense against inflammation and oxidative stress [7]. In the context of T2D, HO-1 has been reported to exhibit protective effects in several experimental models of T2D, including ob/ob mice and diabetic rats. For example, chronic induction of HO-1 improved insulin sensitivity and glucose intolerance and these benefits were attributed to its anti-inflammatory and adipogenic effects [11, 12, 26]. Furthermore, HO-1 activation has been reported to directly regulate insulin signaling in insulin responsive tissues, such as, skeletal muscle, liver, and adipose tissues via PI3K/Akt pathway activation [27]. In several *in vitro* studies, baicalein was found to protect macrophages and cardiomyocytes from oxidative stress and to induce HO-1 expression [18, 19, 28]. However, although baicalein has been reported to induce HO-1 expression in various cells, the molecular association between its HO-1 induction and antidiabetic activities has not been studied. The present study shows the protective effects of baicalein against PA-induced lipotoxicity in β-cells are mediated by HO-1 induction, as both HO-1 inhibitor (ZnPP) and HO-1 siRNA blocked the protective effects of baicalein against PA-induced lipotoxicity.

MAPKs, such as, ERK1/2, p38 MAPK, and JNK, are key players in the expressional up-regulation of Nrf-2-dependent HO-1 [29, 30], and it has also been reported that ERK activation by baicalein protected macrophages from oxidant-induced apoptosis via a HO-1 dependent mechanism [18]. In the present study, baicalein treatment led to the phosphorylation of ERK1/2 and the subsequent induction of HO-1. Furthermore, pharmacological inhibition of ERK1/2 using its specific inhibitor PD98059, abolished the up-regulation of HO-1 by baicalein, indicating that ERK is the upstream regulator of HO-1. In line with this observation, PD98059 pretreatment blocked the beneficial effects of baicalein on ER stress and inflammatory markers. On the other hand, neither SP600125 nor SB203580 showed any effect on PA-induced lipotoxicity (results not shown). Together, these results imply that the protective effect of baicalein against PA-induced lipotoxicity was mediated through ERK-HO-1 cellular defensive activity, which is known to be a critical signal for β-cell function [31]. Although some controversy exists regarding the beneficial effects of HO-1 induction on diabetes [12, 32, 33], our results provide evidence that HO-1 acts as a positive mediator of the action of baicalein on PA-induced β-cell dysfunction. However, it is also possible that additional mechanisms contribute to this protective effect of baicalein. For example, baicalein has been shown to inhibit 12-lipoxygenase (12-LO), which may induce pancreatic β-cell death [34, 35], and long term baicalein administration potently suppressed fatty acid synthesis, gluconeogenesis, and pro-inflammatory responses in hepatocytes by activating AMPK and prevented human islet death by inhibiting the 12-LO pathway [23, 36]. Therefore, we suggest further studies be conducted to identify the action mechanisms of baicalein that contribute to its anti-diabetic effects.

The clinical implications of HO-1 induction in diabetes are debatable, for example, the extent of HO-1 induction varies due to the presence of a microsatellite polymorphism in HO-1 promoter, which could cause unpredictable therapeutic effects [32]. Surprisingly, a recent study of matched biopsies from healthy versus insulin resistant obese human subjects concluded HO-1 is a strong positive indicator of metabolic disease [12]. Furthermore, HO-1 deficiency was found to promote insulin sensitivity and reduce diet-induced fatty liver disease [12, 33], which suggests a novel or additional role for HO-1 in the metabolic regulation of cellular signaling thresholds. In the present study, PA slightly induced HO-1 expression, which could indicate PA has detrimental effects. On the other hand, this induction could also reflect the presence of a compensatory mechanism initiated to manage PA-induced lipotoxicity. Our previous finding that sodium nitroprusside (SNP; a nitric oxide (NO) producer) induced HO-1 to protect SNP-induced apoptosis in the vascular smooth muscle cells (VSMCs) supports this latter explanation [37].

CO is one of the metabolites generated by the catalytic action of HO-1 and has been reported to have several effects on diabetes and obesity. In high fat diet-induced mice, CO inhalation at > 200 ppm or treatment with RuCO, which releases carbon monoxide, prevented weight gain and lowered blood glucose levels [38]. The effects of CO on β-cell dysfunction are under investigation. Recent studies have shown that pharmacological application of CO ameliorates β-cell dysfunction in type 1 diabetes [39, 40]. Similarly, we observed RuCO had beneficial effects on PA-induced lipotoxicity in INS-1 cells, for example, it reduced ER stress. The mechanisms underlying the effects of CO and the possible involvements of bilirubin and iron in β-cell function are currently under investigation.

In summary, the present study indicates baicalein protects pancreatic β-cells from PA-induced lipotoxicity, at least in part, via an ERK-HO-1 pathway. Furthermore, the study shows baicalein has clinical potential as a treatment to improve β-cell dysfunction in diabetes.
